# Increased Abundance and Nursery Habitat Use of the Bull Shark (*Carcharhinus leucas*) in Response to a Changing Environment in a Warm-Temperate Estuary

**DOI:** 10.1038/s41598-018-24510-z

**Published:** 2018-04-16

**Authors:** Charles W. Bangley, Lee Paramore, David S. Shiffman, Roger A. Rulifson

**Affiliations:** 1 Institute for Coastal Science and Policy, East Carolina University, East 5th St, Greenville, North Carolina 27858 USA; 2 North Carolina Division of Marine Fisheries, Manteo Field Office, 1021 Driftwood Drive, Manteo, North Carolina 27954 USA; 3 Earth to Oceans Group, Department of Biological Sciences, Simon Fraser University, 8888 University Drive, Burnaby, BC V5A 1S6 Canada; 4 Fish and Invertebrate Ecology Laboratory, Smithsonian Environmental Research Center, 647 Contees Wharf Road, Edgewater, Maryland 21037 USA

## Abstract

A general northward shift in marine species distributions has been observed in the western North Atlantic Ocean, which may have significant ecological consequences. Large coastal sharks can have wide migratory distributions but show fidelity to specific nursery habitats. Here we show evidence for nursery range expansion into Pamlico Sound, North Carolina by a marine apex predator, the Bull Shark (*Carcharhinus leucas*). Previous assessments have shown little to no use of estuarine North Carolina waters as nursery habitat by Bull Sharks from 1965–2011. Juvenile sharks were rarely captured in a fishery-independent gillnet survey conducted by the North Carolina Division of Marine Fisheries (NCDMF) from 2003–2011, but were present every year from 2011–2016. Juvenile Bull Shark presence in the Sound was strongly related to early summer temperatures and late summer salinities, which have increased in the estuary over the 13 survey years, and further evidence for increasing water temperatures in Pamlico Sound was found in a 45-year data set for the NCDMF estuarine trawl survey. These results suggest that increasing water temperature and salinity have allowed Bull Sharks to expand their nursery habitat. This shift will have unknown, but potentially strong, impacts on both the local ecosystem and interactions with humans.

## Introduction

As ocean temperatures increase, marine taxa may shift their distributions poleward to remain within optimal temperature ranges^1,2^. This trend has been observed in hundreds of marine taxa^3,4^. For example, on the east coast of the United States, Cape Hatteras, North Carolina is positioned at a transition zone between temperate and subtropical marine ecosystems^5,6^ where a local increase in species typically associated with more tropical waters has been observed in recent years^7^.

Shifts in biodiversity can result in significant changes to local food web dynamics, ecosystem productivity, and even fisheries^2^. These ecological effects may be even more pronounced if a species new to a region is an apex predator, such as a shark^8^. Sharks are highly mobile and many species are highly migratory, which makes them among the marine taxa most likely to shift distribution in response to climate change^9^. However, species with restricted ranges or associations with specific habitat areas may be more vulnerable^10^.

Many species of shark rely on shallow coastal or estuarine nursery areas during early life stages^11^. Nursery areas for the Bull Shark (*Carcharhinus leucas*) have been documented in the northern Gulf of Mexico^12–15^ and the east coast of Florida^16,17^. Extensive surveys of shark nursery habitats along the U.S. east coast have found little evidence of Bull Shark nursery habitat north of Indian River Lagoon, Florida^18–20^. A previous study compiling fishery-independent survey data, fishery landings, and sightings of Bull Shark in the coastal and estuarine waters of North Carolina documented 113 individuals from 1965–2011, but only nine were within juvenile size range^20^.

Bull Shark nursery habitats tend to be located in areas with limited access to the ocean and highly variable salinity, often associated with barrier islands and narrow inlets^12,14,15,17,21,22^. Pamlico Sound, North Carolina shares many of these features with known Bull Shark nurseries^23^, suggesting that the historical rarity of juvenile Bull Shark in this system^20,24^ may be due to other factors such as temperature preferences or natal philopatry to other estuaries^25^. Here we show evidence for recent changes in environmental conditions in the estuary that may be associated with an expansion of Bull Shark nursery habitat into Pamlico Sound.

## Results

A total of 70 Bull Sharks were captured from 2003–2016 in the NCDMF gillnet survey. Based on known size-at-age ranges^26^, 28 Bull Sharks were classified as Young-of-Year (YOY), 33 as Age 1+, and length was not recorded for nine individuals. Sharks without recorded length were excluded from further analysis due to the inability to confirm age class for these individuals.

**Figure 1:**
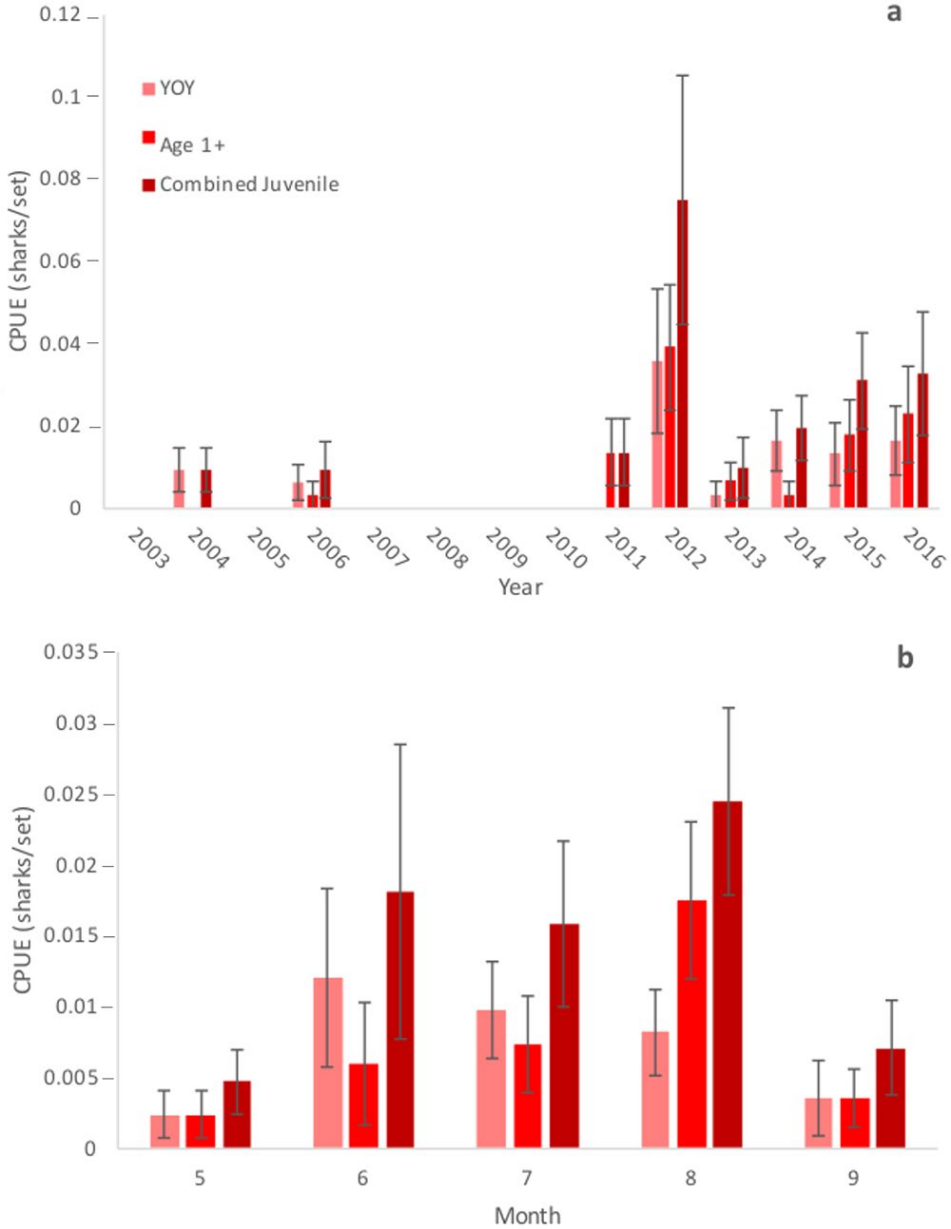
Catch per unit effort (CPUE, sharks/set) of Young-of-Year (YOY), Age 1+, and Combined Juvenile size classes during May-September in 2003–2016 NCDMF fishery-independent gillnet surveys within Pamlico Sound by (**a**) year and (**b**) month. Error bars represent standard error.

Bull Sharks in juvenile size classes were not consistently captured prior to 2011 but were captured in low numbers in 2004 and 2006 (Fig. 1a). YOY and Age 1+ Bull Shark catch per unit effort (CPUE) was greatest during 2012 and sharks within juvenile size ranges made up the entirety of the catch in every subsequent year. CPUE declined sharply in 2013 but increased and remained relatively consistent in the following years (Fig. 1a). Juvenile Bull Sharks were recorded from May-September, with the majority of catches occurring June-August (Fig. 1b). Total length showed increasing relationships with month (*F* = 9.576, *p* = 0.003, df = 1) and year (*F* = 6.423, *p* = 0.014, df = 1) (Supplementary Fig. S1). Bull Sharks within a neonate length range were captured from May-July (Supplementary Fig. S1a).

**Figure 2:**
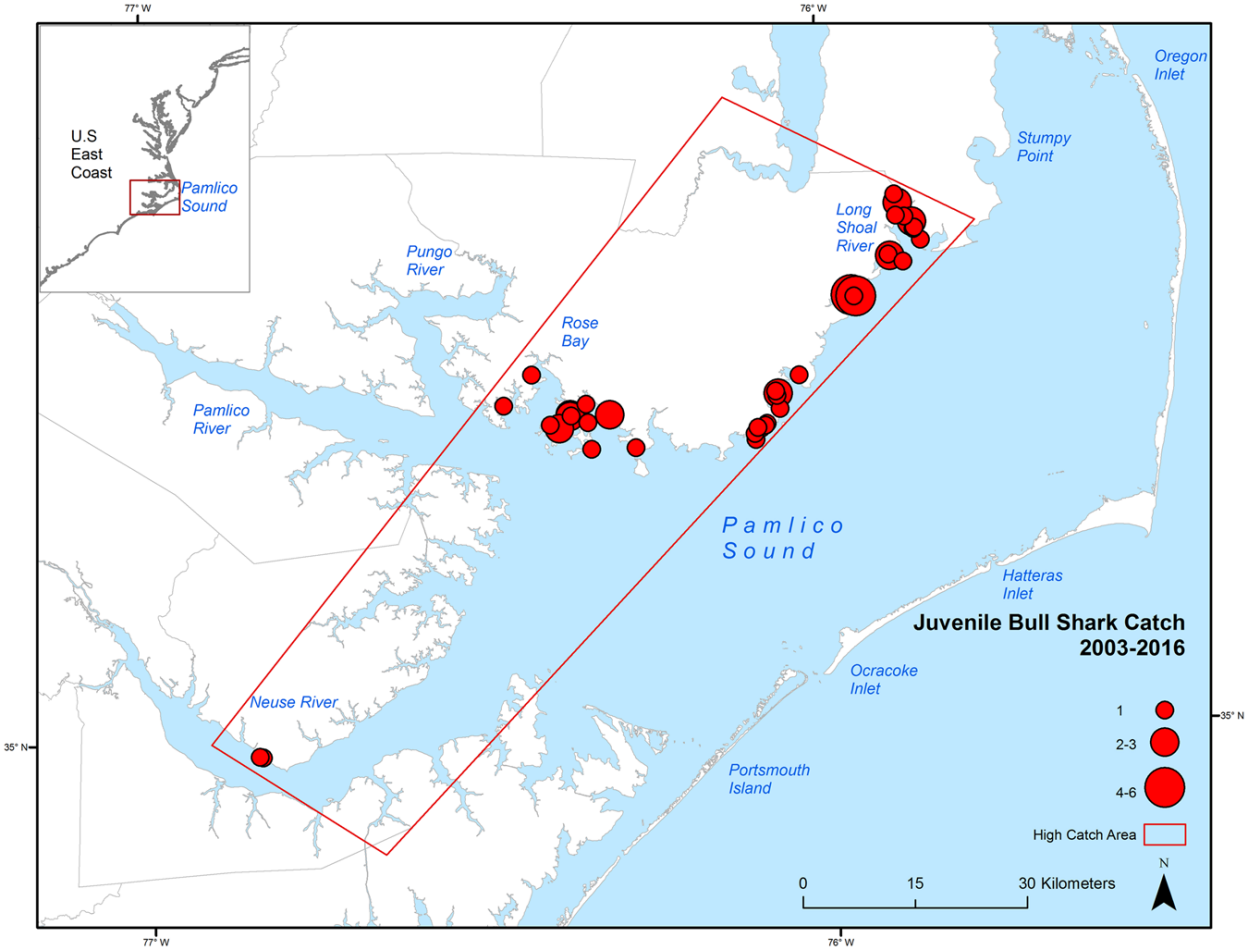
Capture locations and total catch (N sharks) of juvenile Bull Sharks (Young-of-Year and Age 1+ combined) in sets from the North Carolina Division of Marine Fisheries (NCDMF) fishery-independent gillnet survey within Pamlico Sound. Data are from the 2003–2016 survey years. The polygon represents the border of the high catch area for juvenile Bull Sharks in the survey. Map created using ArcGIS 10.4 (ESRI, Inc. Redland, CA, USA http://desktop.arcgis.com/en/).

Catches of juvenile Bull Sharks were spatially distributed along the western shore of Pamlico Sound from the Long Shoal River to Rose Bay, and within the lower Neuse River (Fig. 2). This area, and a portion of the lower Neuse River, were included within the polygon designating the high catch area for juvenile Bull Sharks in the survey. The greatest numbers were captured in the vicinity of the Long Shoal River and lower Rose Bay (Fig. 2). No significant relationships were found between year and latitude (*F* = 1.07, *p* = 0.301, df = 1) or longitude (*F* = 2.34, *p = *0.126, df = 1) of survey stations.

Bull Sharks were captured across a range of environmental gradients. One-way ANOVA results showed significant differences in mean temperature and inlet distance between life stages (Table 1). Age 1+ sharks occurred at a higher mean temperature and a greater mean inlet distance than YOY sharks (Table 1). No Bull Sharks in juvenile size classes were captured at temperatures below 22 °C.

**Table 1:** Mean ( ± standard deviation) of environmental and spatial variables measured at stations where each Bull Shark life stage was captured by the NCDMF gillnet survey in Pamlico Sound, with ANOVA results (DF = 2) determining significant differences between life stages.

Life Stage	Depth (m)	Temperature (°C)	Salinity (ppt)	Dissolved O_2_ (mg/L)	SAV Distance (m)	Inlet Distance (km)
YOY	1.40 ± 0.57	27.94 ± 2.28	18.18 ± 4.00	6.81 ± 1.33	1201.23 ± 931.70	38.92 ± 4.96
Age 1+	1.33 ± 0.51	29.44 ± 1.73	16.94 ± 4.27	6.52 ± 1.22	1175.05 ± 1135.54	41.90 ± 5.75
F	0.213	6.185	1.172	0.606	0.009	4.182
p	0.646	0.016	0.283	0.439	0.926	0.04

Because YOY and Age 1+ Bull Sharks could be distinguished based on environmental variables, generalized linear model (GLM) analysis was conducted on YOY and Age 1+ sharks independently, and on both age classes grouped together as Combined Juveniles. Inlet distance and year were dropped from both the whole sound and high catch area models due to collinearity with other variables. For the whole sound, the model with the lowest AICc value included all other variables. Only temperature and salinity were included in the final high catch area model, which had a lower AICc value than the whole sound model. Zero-inflated negative binomial-distributed GLM results showed that temperature and salinity had significant effects on CPUE for all age classes in both models (Table 2). Zero-inflation was not significantly related to any variables for Age 1+ and Combined Juveniles but was did have significant relationships with salinity and SAV distance among YOY sharks in the whole sound model (Table 2). The high catch area model was selected for use predicting CPUE (Whole sound model AICc = 445.78, high catch area model AICc = 412.57). Plots of juvenile Bull Shark CPUE against significant environmental variables suggested positive associations with temperatures greater than 22 °C and salinities between 10 and 25 ppt (Supplementary Fig. S2).

**Table 2:** Effect size (*z*-score) and significance (*p*) for zero-inflated negative binomial-distributed generalized linear models relating CPUE (sharks/set) of Bull Sharks in each life stage with year and environmental and spatial variables from the NCDMF gillnet survey of Pamlico Sound, NC in both the whole sound and high catch area.

Whole Sound	GLM	Combined Juvenile		YOY		Age 1+	
Variable		CPUE	Zero-Inflation	CPUE	Zero-Inflation	CPUE	Zero-Inflation
Depth	z	0.751	1.021	1.631	1.436	0.765	0.612
	p	0.453	0.307	0.103	0.151	0.444	0.541
Temp	z	**3**.**826**	1.067	**3**.**124**	1.812	**3**.**338**	1.066
	p	**<0**.**001**	0.286	**0**.**002**	0.07	**<0**.**001**	0.286
Sal	z	**6**.**339**	1.099	**6**.**767**	**3**.**522**	**5**.**656**	0.98
	p	**<0**.**001**	0.272	**<0**.**001**	**<0**.**001**	**<0**.**001**	0.327
DO	z	−0.746	1.074	−672	−1.124	−0.52	0.017
	p	0.455	0.283	0.501	0.901	0.603	0.986
SAV Dist	z	N/A	−1.05	N/A	−15.065	N/A	−0.711
	p	N/A	0.294	N/A	**<**0.001	N/A	0.477
**High Catch Area**	**GLM**	**Combined Juvenile**		**YOY**		**Age1+**	
**Variable**		**CPUE**	**Zero-Inflation**	**CPUE**	**Zero-Inflation**	**CPUE**	**Zero-Inflation**
Temp	z	**2**.**841**	1.478	1.909	0.749	**2**.**561**	1.134
	p	**0**.**005**	0.139	0.056	0.454	**0**.**01**	0.257
Sal	z	**3**.**394**	1.608	**4**.**713**	2.29	**2**.**897**	1.617
	p	**<0**.**001**	0.108	**<0**.**001**	0.022	**0**.**003**	0.106

Significant relationships (α = 0.05) are in bold.

**Figure 3:**
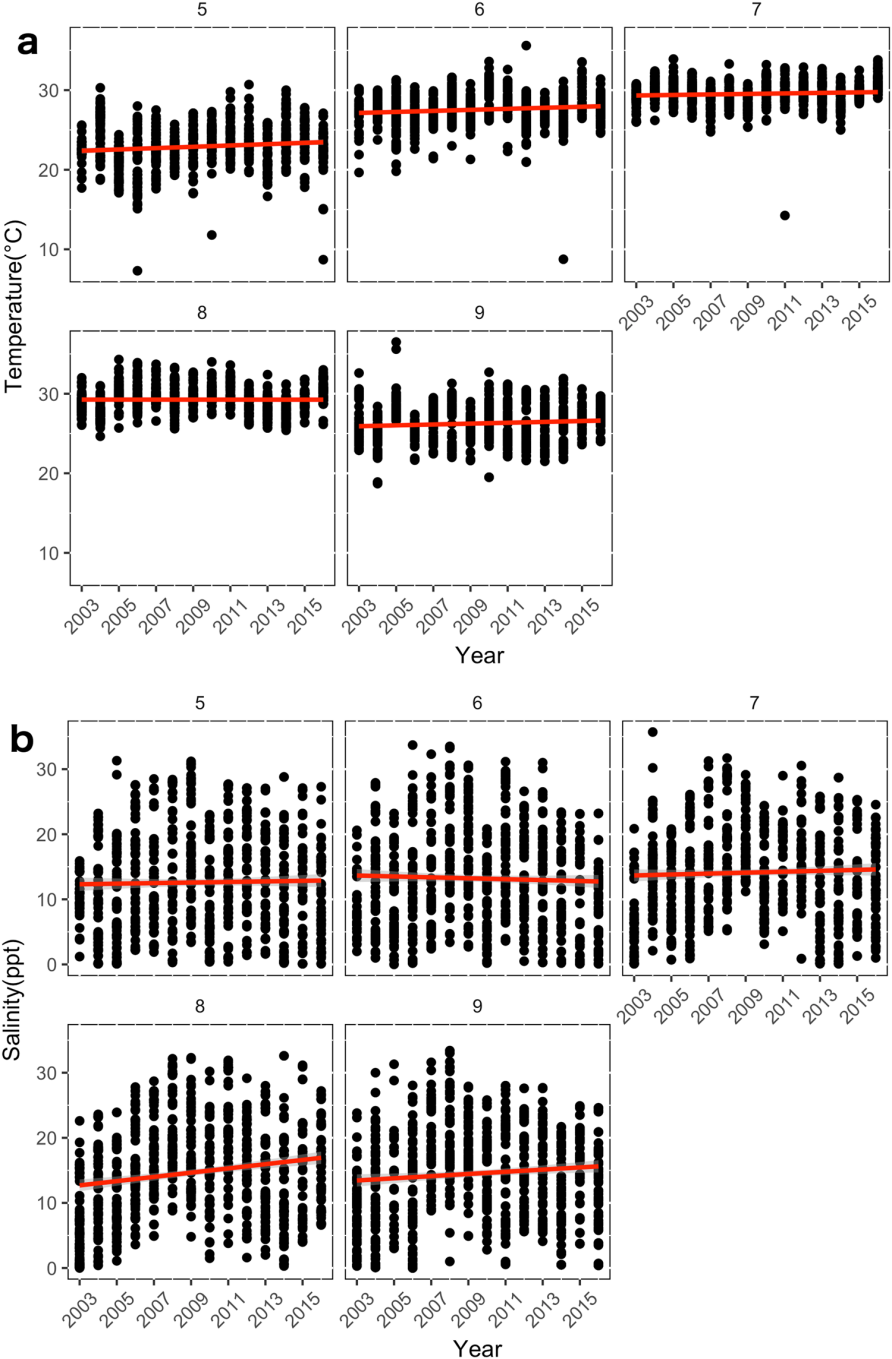
Linear trends of May-September (months 5–9) (**a**) water temperature (°C) and (**b**) salinity (ppt) measurements by year recorded by the NCDMF fishery-independent gillnet survey. Data are from 2003–2016 survey years.

Temporal trends in environmental variables that were significantly related to Bull Shark CPUE varied by month (Fig. 3, Table 3). Linear regression showed that temperature increased over 2003–2016 by approximately 0.08 °C/year in May, 0.06 °C/year in June, 0.03 °C/year in July, and 0.05 °C/year in September. Salinity increased over the same period by approximately 0.32 ppt/year in August and 0.17 ppt/year in September. The best-fitting relationships were temperature increases during May and June and salinity increases during August and September (Fig. 3, Table 3).

**Table 3:** Initial measurements (mean ± standard deviation) from the 2003 survey year and results of linear regression analysis of variables found to have a significant relationship with Combined Juvenile Bull Shark CPUE over Year, grouped by month.

Month		Temperature (°C)	Salinity (ppt)
May	2003 Mean ± SD	**22**.**34 ± 1**.**68**	10.28 ± 3.40
	Estimate	**0**.**08 ± 0**.**02**	0.04 ± 0.07
	p	**<0**.**001**	0.522
	r^2^	**0**.**013**	0.0004
June	2003 Mean ± SD	**26**.**76 ± 2**.**27**	9.77 ± 4.95
	Estimate	**0**.**06 ± 0**.**02**	−0.07 ± 0.06
	p	**<0**.**001**	0.297
	r^2^	**0**.**013**	0.001
July	2003 Mean ± SD	**28**.**93 ± 1**.**33**	7.90 ± 5.91
	Estimate	**0**.**03 ± 0**.**01**	0.07 ± 0.06
	p	**0**.**022**	0.26
	r^2^	**0**.**005**	0.0003
August	2003 Mean ± SD	28.67 ± 1.27	**6**.**64 ± 5**.**87**
	Estimate	−0.01 ± 0.01	**0**.**32 ± 0**.**06**
	p	0.956	**<0**.**001**
	r^2^	0.001	**0**.**031**
September	2003 Mean ± SD	**25**.**99 ± 2**.**33**	**9**.**00 ± 5**.**68**
	Estimate	**0**.**05 ± 0**.**02**	**0**.**17 ± 0**.**06**
	p	**0**.**006**	**0**.**008**
	r^2^	**0**.**007**	**0**.**007**

**Figure 4:**
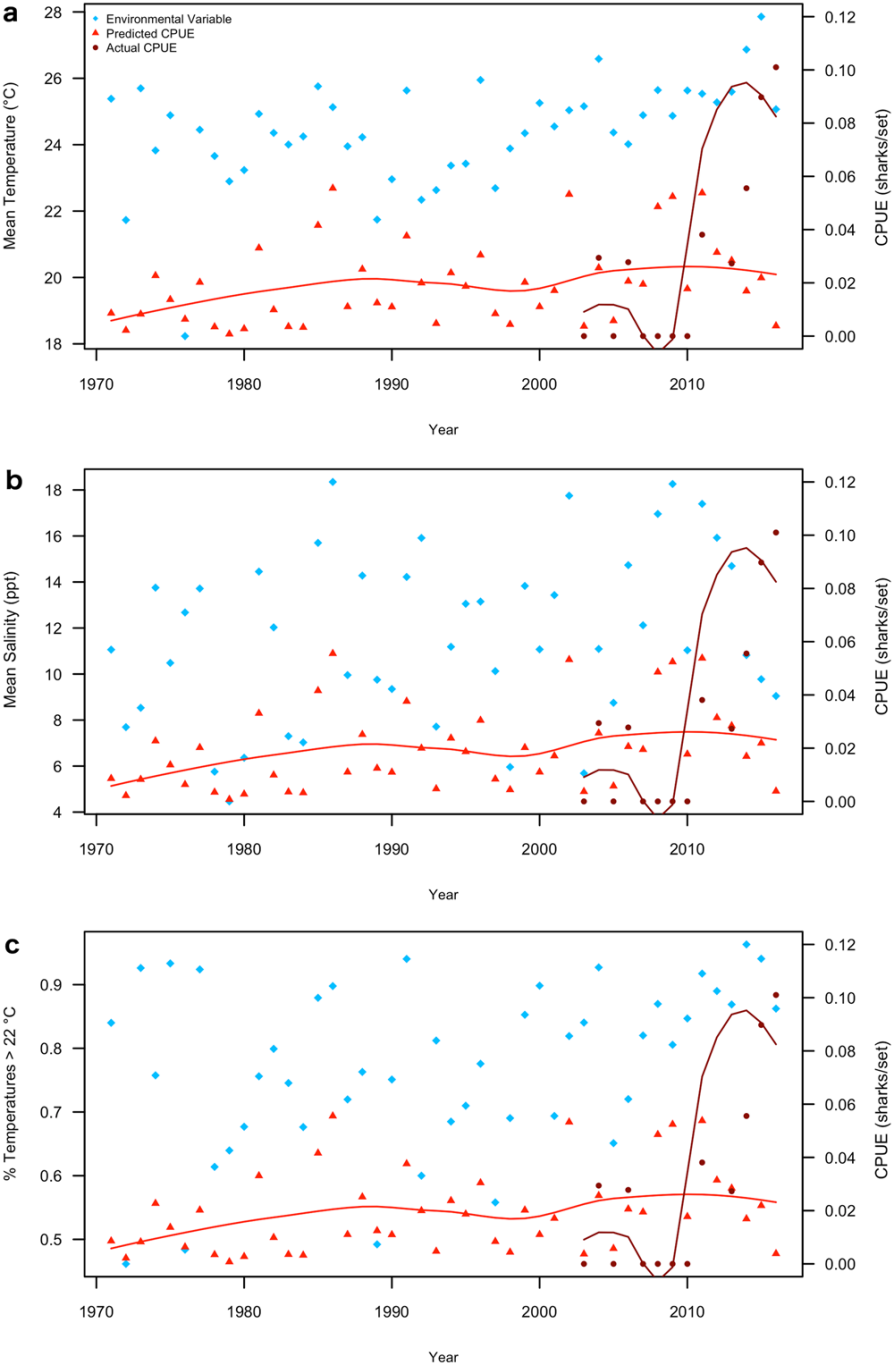
Annual mean May-June (**a**) temperatures (°C), (**b**) salinities (ppt), and (**c**) percentage of temperatures above the minimum at which Bull Sharks were present (22 °C) from the North Carolina Division of Marine Fisheries (NCDMF) Pamlico Sound estuarine trawl survey with actual annual mean juvenile Bull Shark CPUE from the NCDMF gillnet survey and predicted mean CPUE from the zero-inflated negative binomial GLM applied to environmental data from the trawl survey. Predicted and actual CPUE data are from the high catch area of the NCDMF gillnet survey, and Loess lines represent smoothed CPUE trends.

Linear regression analysis of 1971–2016 May NCDMF trawl survey data showed significant increasing trends for temperature, salinity, and percentage of above-minimum temperatures, with percentage of above-minimum temperatures providing showing the best fit (Table 4). CPUE was predicted from trawl survey data using the GLM from the high catch area, which included only temperature and salinity. Predicted CPUE steadily increased over the trawl survey period but did not show the dramatic increase in 2011 found in the empirical data (Fig. 4).

**Table 4:** Results of linear regression analysis of variables found to have a significant relationship with Combined Juvenile Bull Shark CPUE over Year using data recorded during the NCDMF Juvenile Estuarine Trawl Survey.

Variable	Estimate	SE	*t*	*p*	r^2^
Temp	0.049	0.004	13.010	**<**0.001	0.016
Sal	0.092	0.005	19.170	**<**0.001	0.033
% Temp > 22 °C	0.003	0.001	2.558	0.014	0.110

Temperature metrics more closely followed actual CPUE, with 2008 appearing to mark a shift after which no mean temperatures below 25 °C occurred (Fig. 4a) and over 60% of temperatures fell above the minimum for Bull Shark presence (Fig. 4c). Mean salinity was considerably more variable but mean salinities less than 9 ppt did not occur after 2005 (Fig. 4b).

## Discussion

To our knowledge, this study is the first to attempt to associate environmental factors with the apparent colonization of a nursery habitat by a marine apex predator. Our results provide strong evidence that an increase in the use of Pamlico Sound as a Bull Shark nursery is associated with changing environmental conditions in the estuary, with temperature as the likely primary driver of Bull Shark presence. Though Bull Sharks were a rare catch overall in NCDMF surveys, the number of juveniles captured in Pamlico Sound increased from six from 2003–2011 (average of 0.75 sharks per year) to 53 captured from 2011–2016 (average of 8.8 sharks per year) with a corresponding increase in CPUE. This represents a dramatic increase in juvenile Bull Shark presence from previous assessments of shark nursery habitat use in North Carolina waters^20,24^. Though 38.9% of the juvenile Bull Sharks in the survey were caught in 2012, their presence became more consistent beginning in 2011, with Age 1+ sharks caught every year and YOY sharks captured every year since 2012. A steady increase in juvenile Bull Shark CPUE was predicted when using the model generated with gillnet survey data to predict CPUE based on environmental measurements taken during the trawl survey, showing that at least two independent surveys have documented changing conditions in the estuary that are positively associated with shark abundance.

We found that the increased CPUE of juvenile Bull Sharks was most strongly associated with increasing water temperatures and salinities, which occurred in early and late summer, respectively. These environmental changes were consistent between both gillnet and trawl survey data, suggesting that increasing early summer temperatures and late summer salinities have been consistent trends in this system. While salinity was highly variable year-to-year, the increasing trend in water temperature became more consistent in trawl survey data after 2008. Generally, water temperature influences both large-scale seasonal migration and finer-scale habitat selection in sharks, while salinity is more strongly associated with localized movements and habitat use^27^. In nursery habitats along coastal Florida, neonate and YOY Bull Sharks occupy a narrower salinity range than other demographic groups^17^ and move within estuaries to follow preferred salinity gradients^13^. However, Bull Sharks were not present at all at temperatures lower than 18–20 °C in the Indian River Lagoon^17^. Juvenile Bull Sharks remain within their nursery estuaries year-round, only leaving during cold temperature events, which can also cause cold stuns and mortality^17,28^. Our data showed that increased CPUE of juvenile-sized Bull Sharks in Pamlico Sound associated with a minimum temperature of approximately 22 °C and greater than 9 ppt mean salinity. Pamlico Sound temperatures are considerably lower than this threshold during the winter and no Bull Sharks were captured in the survey from October – April, so it likely that juvenile Bull Sharks leave the estuary during this time. Therefore, the seasonal presence and possibly birth of juvenile Bull Sharks may be associated with temperature, while salinity likely influences spatial distribution within the estuary. However, the lack of a sharp increase in 2011 in the model-predicted CPUE suggests that there may be some other cause for the initial sudden increase in shark CPUE during this time, potentially a partruition or immigration event not directly associated with the environmental variables we analyzed.

In comparison with other areas of the U.S. Atlantic coast, northwestern Pamlico Sound represents the most likely nursery habitat north of the Indian River Lagoon based on the criteria of Heupel *et al*.^11^: juveniles are more commonly encountered there than in other southeastern U.S. estuaries^18^ or parts of Pamlico Sound, are present in the system for the majority of the summer, and since 2011 have been documented every year. Bull Shark parturition occurs from May-August in the Indian River Lagoon^16,17^, which coincides with the appearance of the smallest YOY Bull Sharks in Pamlico Sound. Previously, the Indian River Lagoon system in Florida was thought to be the northernmost functional Bull Shark nursery on the U.S. Atlantic coast^17^. However, sporadic captures of neonate/YOY Bull Sharks in estuaries in Georgia and the Carolinas^19,24,29^ suggest that parturition may occur in areas north of the Indian River Lagoon on a limited, opportunistic basis if conditions allow, though studies with longer time series in these areas have not shown a consistent annual presence of juveniles. Captures of YOY Bull Sharks in 2004 and 2006 may represent opportunistic pupping during years with favorable conditions, while the consistent presence of juveniles since 2011 may be a result of consistently warm late spring/early summer temperatures allowing sharks previously born in Pamlico Sound to return. The size range of juveniles has also expanded to include larger individuals in the most recent survey years, which is further evidence of presence over multiple years. Bull Sharks show evidence of strong philopatry and both pregnant females and juveniles are likely to repeatedly return to primary nurseries^25^, though if colonization of Pamlico Sound as a nursery is occurring then this would strongly suggest that straying is possible. The use of Pamlico Sound as a primary nursery may increase further if water temperatures remain warm and females born in the system reach maturity and return to give birth. The repeated return of individuals will need to be directly assessed to truly determine whether Bull Sharks are showing site fidelity or philopatry to Pamlico Sound, so the use of genetic and telemetry methods to verify repeated annual presence of individuals are necessary next steps.

The increase in juvenile Bull Shark abundance in Pamlico Sound coincides with a general northward shift in marine species distributions along the U.S. Atlantic coast^4,30,31^. At least one other large shark, the Sand Tiger Shark (*Carcharias taurus*), has recently been observed using areas where it was historically rare as nursery habitat^32^. There is evidence that Bull Shark populations are recovering from previous declines^33,34^, which may cause apparent range expansion as the species returns to parts of its historical range, but the lack of evidence for nursery habitat in Pamlico Sound extends to years prior to documented shark population declines in U.S. Atlantic waters^20^. Another possible explanation is a currently undescribed decadal-scale trend in Bull Shark population dynamics similar to one recently discovered among Lemon Sharks (*Negaprion brevirostris*) in Bimini^35^. Such a trend could not be inferred from the only long-term study on Bull Shark catches in North Carolina waters due to a lack of standardization between survey and observational data^20^. Changes in the catch of all shark species combined have been observed in the University of North Carolina shark survey near Cape Lookout, North Carolina but factors causing or influencing these changes are unclear^36^. With limited evidence for other explanations, we conclude that the increase in juvenile Bull Shark abundance in Pamlico Sound is related to increasing water temperatures and salnities during the time of parturition.

Many wide-ranging large coastal sharks depend on specific regions or sites as nursery habitat^37^, and changing environmental conditions at these sites may have a disproportionate effect on their populations^3^. Our results suggest that Bull Sharks have the potential to colonize new nursery habitats, which may make their populations resilient to large-scale disturbances such as climate change. A local increase in Bull Shark presence may exert new top-down effects on Pamlico Sound communities^8^, but interspecific interactions involving Bull Sharks in this estuary have not been assessed. Additionally, Bull Sharks are considered dangerous to humans^38^ and have been implicated in depredations on species targeted by recreational fisheries within North Carolina estuaries (Bangley, personal observation), so localized population increases may affect the frequency and nature of local human-shark interactions. Further monitoring will be needed to determine the ecological effects of Bull Shark nursery expansion into Pamlico Sound and to predict further range expansion.

## Methods

**Figure 5:**
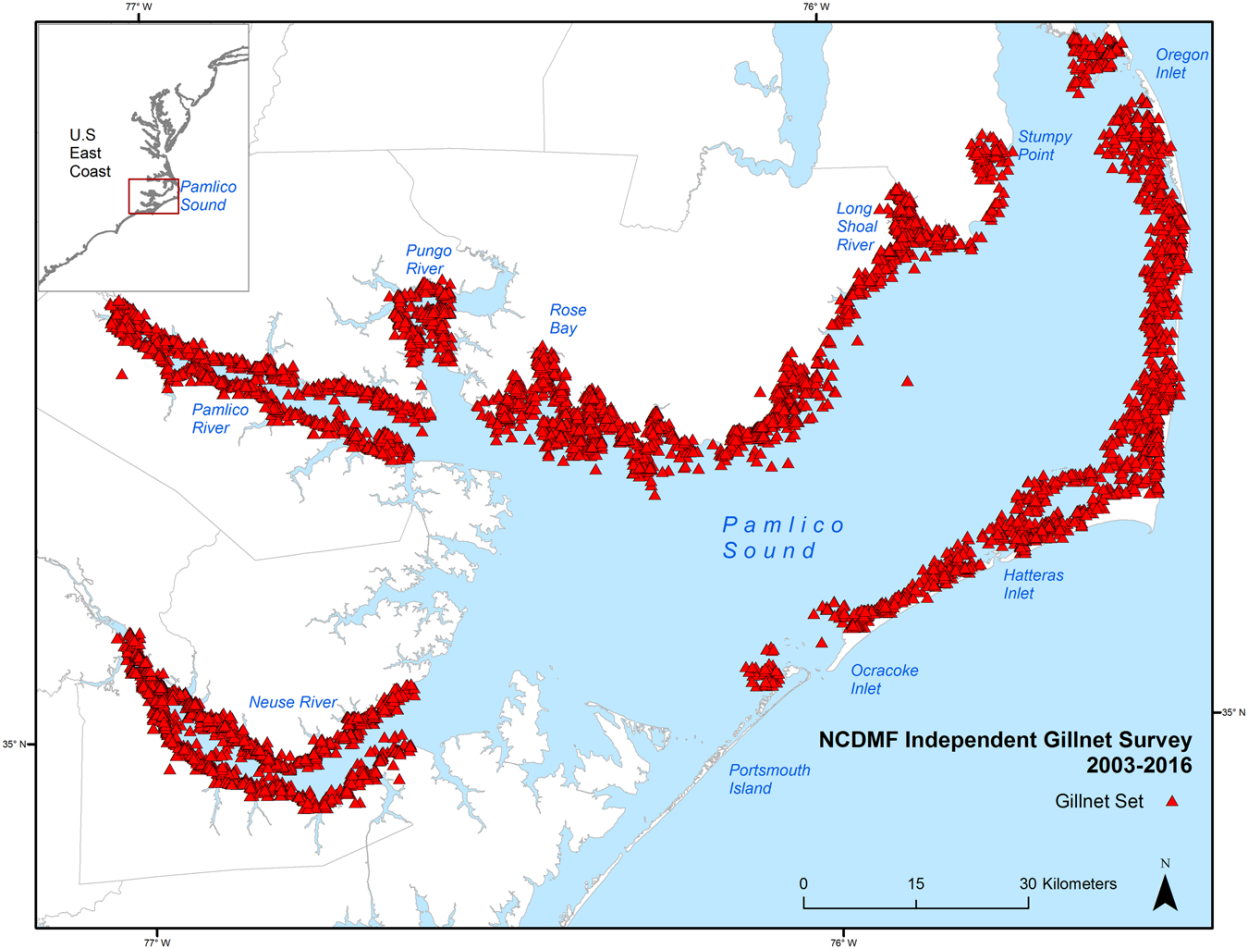
Sampling locations for years 2003–2015 of the North Carolina Division of Marine Fisheries (NCDMF) fishery-independent gillnet survey within Pamlico Sound. Map created using ArcGIS 10.4 (ESRI, Inc. Redland, CA, USA http://desktop.arcgis.com/en/).

Environmental and Bull Shark catch data from the North Carolina Division of Marine Fisheries (NCMDF) independent gillnet survey were analyzed for years 2003–2016. The gillnet survey has been conducted since 2001 but was expanded to include stations in the Neuse and Pamlico Rivers in 2003, so only data collected since 2003 were used to account for potential shark captures in lower salinities. The total study area for the gillnet survey ranged from Oregon Inlet to Portsmouth Island on the east side of Pamlico Sound and from Stumpy Point to Rose Bay on the western side, and included the estuarine portions of the Neuse, Pamlico, and Pungo Rivers (Fig. 5). The study area was divided into 1.85-km by 1.85-km cells, and sampling stations were selected by randomly choosing grid cells prior to sampling. The gillnet survey was conducted from February-December, and utilized a 219.46-m sink gillnet constructed of eight 27.43-m sections of 7.62-, 8.89-, 10.16-, 11.43-, 12.70-, 13.97-, 15.24-, and 16.51-cm stretched monofilament mesh soaked for 12 hours from sunset to sunrise. Sampling effort ranged from 487–645 sets per year (Supplementary Table S1). Depth (m), water temperature (°C), salinity (ppt), and dissolved oxygen (mg/L) were recorded at each sampling station, and distance from the station to the nearest inlet (inlet distance, km) and the nearest mapped submerged aquatic vegetation (SAV) bed (SAV distance, m) were measured using ArcGIS 10.1. SAV maps were generated using data from aerial and boat-based surveys conducted by the Albemarle-Pamlico National Estuarine Partnership (APNEP)^39^.

All sharks were identified to species, and sex and total length (TL, mm) were recorded. Bull Sharks were classified to life stages based on known total length at age estimates^26^, with sharks 500–810 mm TL classified as young-of-year (YOY), sharks 811–1400 mm TL classified as juveniles one year of age or older (Age 1+), sharks 1401–2000 mm TL classified as subadults, and sharks greater than 2000 mm TL classified as mature adults. Four sharks identified as Bull Sharks but with a recorded TL of less than 500 mm were assumed to be misidentified due to being less than minimum size at birth^24^ and therefore were excluded from further analysis. One-way ANOVA was used to determine significant differences in the measured environmental and spatial variables between Bull Shark life stages, and life stages without any significant differences were combined for analysis.

Bull Shark catch per unit effort (CPUE) was standardized as sharks/set. Possible spatial changes in survey sampling effort were assessed by modeling latitude and longitude over year using Oneway ANOVA. Generalized linear models (GLMs) were used to determine whether year and any environmental or spatial variables were significantly related to Bull Shark CPUE during months when Bull Sharks were captured, and were set at a zero-inflated negative binomial distribution to account for zero-inflation, long-tailed distributions, and overdispersion common in ecological count data. To account for the potential for highly localized environmental variation, two GLMs were generated: one using data from the entire survey area and referred to as the whole sound model, and one only using data from survey sets falling within a polygon drawn around stations where juvenile Bull Sharks were captured and referred to as the high catch area model. In these models, a logit link function was used for the catch data and a log link for zero-inflation. Important environmental variables were identified based on significance (*p*) and effect size (*z*-score). The best model was chosen by initially generating a model with all environmental variables, eliminating variables that would not resolve with the model, and then removing and re-adding the remaining variables in a stepwise fashion using the “step” function in R^40^. The model with the lowest corrected Akaike Correlation Criterion (AICc) was chosen to identify the environmental variables with a significant relationship with Bull Shark CPUE, and to predict CPUE based on those environmental variables. Potential changes over the study period in environmental variables identified as important by GLM results were assessed using linear regression to determine the significance of relationships between environmental measurements and year, during each month Bull Sharks were present.

Long term changes in environmental variables identified by annual trend analysis were assessed using data from the NCDMF fixed-station estuarine trawl survey within Pamlico Sound, which has been conducted annually since 1971 (Supplementary Fig. S3). Depth (m), temperature (°C), salinity (ppt), and dissolved oxygen (mg/L) concentration were recorded at each station. For environmental variables with a significant relationship with juvenile Bull Shark CPUE, linear bivariate ANOVAs were used to determine whether increasing or decreasing trends in those factors were significant over the 45-year period covered by the trawl survey. In addition, the percentage of early summer (May and June) temperatures greater than the minimum temperature at which Bull Sharks were captured in the gillnet survey was calculated for each year and trends were assessed using the same bivariate ANOVA procedure. To determine whether these longer-term changes may have influenced juvenile Bull Shark CPUE, the best GLM generated using the gillnet survey catches was used to predict CPUE based on environmental measurements recorded during the trawl survey. Predicted CPUE was plotted and compared visually with actual CPUE from the gillnet survey and environmental factors.

All statistical analyses were conducted using R statistical software version 3.3.1^40^.

### Ethical Approval

NCDMF is authorized by North Carolina state statute Article 20 § 113–261 to approve all sampling protocols internally. All field sampling procedures followed standard NCDMF-approved protocols.

### Data Availability

North Carolina public data are available through a public records custodian at the applicable agency according to North Carolina state statute (N.C.G.S § 132-2). All NCDMF fishery-independent survey data used in this manuscript can be accessed by contacting Lee Paramore (lee.paramore@ncdenr.gov) and submitting a Biological Data Tracking Form available at http://portal.ncdenr.org/c/document_library/get_file?uuid = 4c762326-de86-4661-a142-31c1d55e49bb&groupId = 38337).

## Electronic supplementary material

Supplementary Information
