# Supplementary material for: Increased Abundance and Nursery Habitat Use of the Bull Shark (Carcharhinus leucas) in Response to a Changing Environment in a Warm-Temperate Estuary
Source: Sci Rep. 2018 Apr 16;8:6018. doi: 10.1038/s41598-018-24510-z (PMC5902476; doi:10.1038/s41598-018-24510-z)
Supplement: Supplementary file 1 — Supplementary Information [file 41598_2018_24510_MOESM1_ESM.docx]

Increased Abundance and Nursery Habitat Use of the Bull Shark (*Carcharhinus leucas*) in Response to a Changing Environment in a Warm-Temperate Estuary

Supplemental Information

Charles W. Bangley^1,4^, Lee Paramore^2^, David S. Shiffman^3^, and Roger A. Rulifson^1^

^1^Institute for Coastal Science and Policy, East Carolina University, East 5^th^ St., Greenville, North Carolina 27858, USA. ^2^North Carolina Division of Marine Fisheries, Manteo Field Office, 1021 Driftwood Drive, Manteo, North Carolina 27954, USA. ^3^Earth to Oceans Group, Department of Biological Sciences, Simon Fraser University, 8888 University Drive, Burnaby, BC, V5A 1S6. ^4^Fish and Invertebrate Ecology Laboratory, Smithsonian Environmental Research Center, 647 Contees Wharf Road, Edgewater, Maryland 21037, USA.

Table S1. North Carolina Division of Marine Fisheries (NCDMF) independent gillnet survey sampling effort (number of sets deployed) in Pamlico Sound, North Carolina per year in the from 2003 through 2015.

| Year | GN |
| --- | --- |
| 2003 | 488 |
| 2004 | 642 |
| 2005 | 608 |
| 2006 | 641 |
| 2007 | 642 |
| 2008 | 643 |
| 2009 | 641 |
| 2010 | 645 |
| 2011 | 619 |
| 2012 | 628 |
| 2013 | 628 |
| 2014 | 628 |
| 2015 | 628 |
| 2016 | 628 |


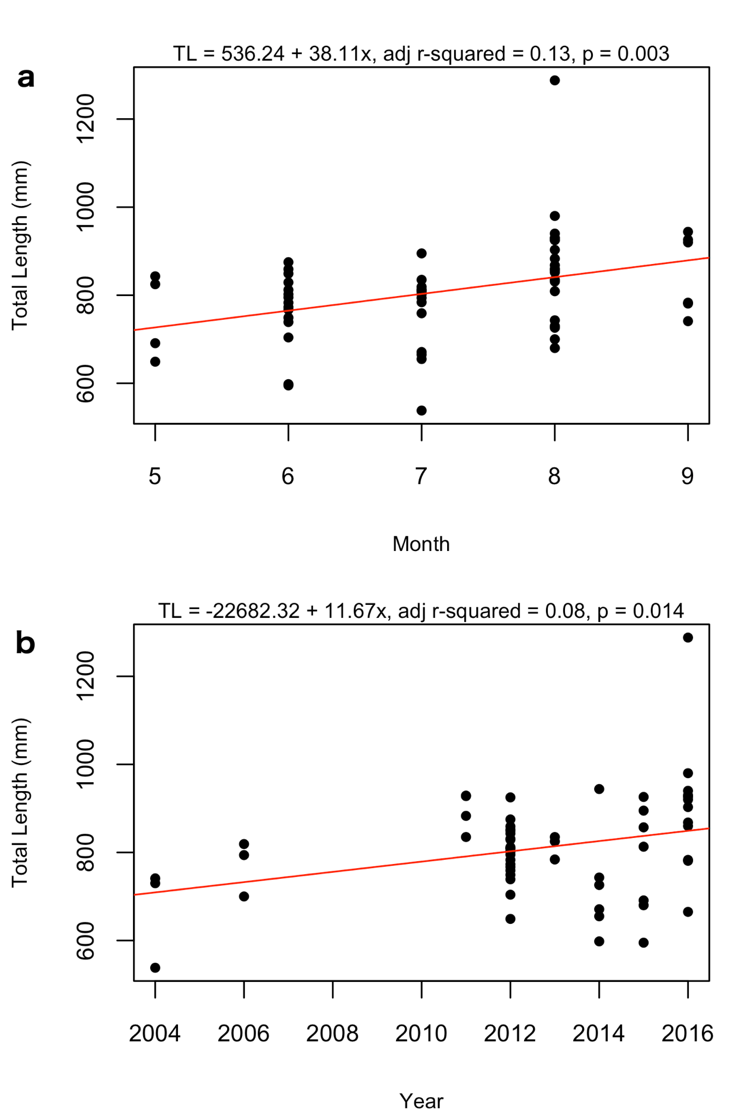


Fig. S1. Linear regression relationship between total length (mm) and (a) month and (b) year of Bull Sharks in captured Pamlico Sound, North Carolina. Sharks were captured in the North Carolina Division of Marine Fisheries (NCDMF) independent gillnet survey during the 2003-2016 survey years.


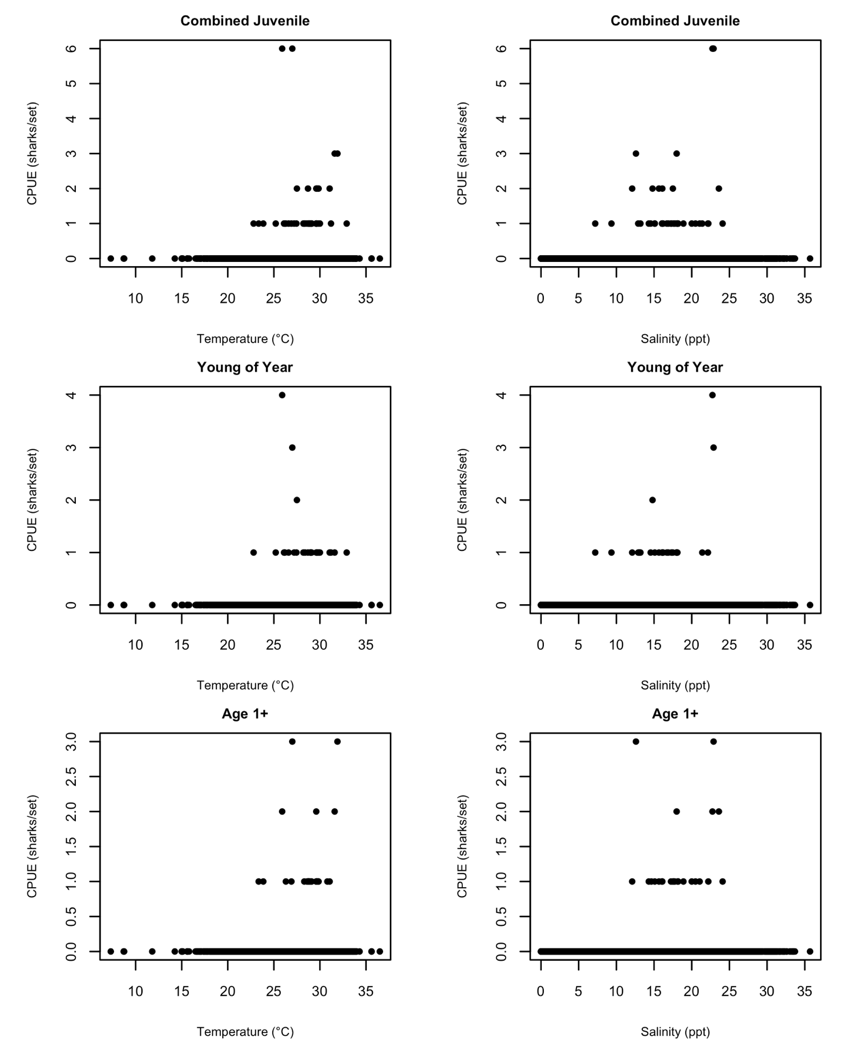


Fig. S2. Bull Shark catch per unit effort (CPUE in sharks/set) in Combined Juvenile (young-of-year and Age 1+ combined), Young of Year, and Age 1+ life stages over environmental variables found to have a significant relationship with CPUE from 2003-2016 NCDMF independent gillnet survey years.


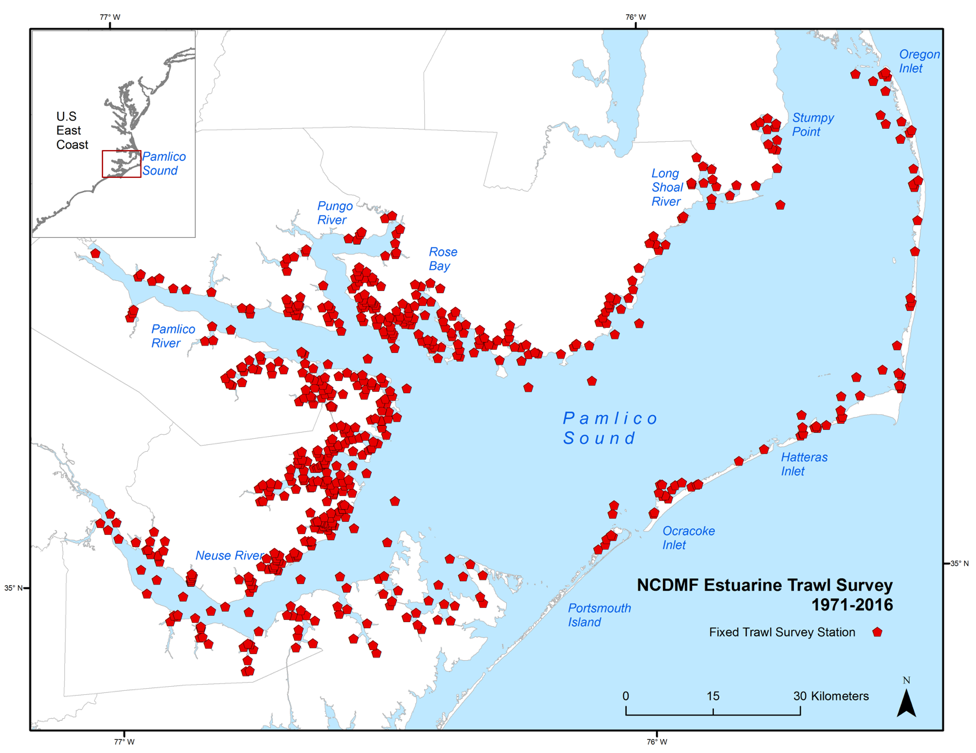


Fig. S3. Locations of fixed stations where environmental data were collected from 1971-2015 North Carolina Division of Marine Fisheries (NCDMF) estuarine trawl surveys within Pamlico Sound. Map created using ArcGIS 10.4 (ESRI, Inc. Redland, CA, USA <http://desktop.arcgis.com/en/)>.
